# The Effects of Manure and Nitrogen Fertilizer Applications on Soil Organic Carbon and Nitrogen in a High-Input Cropping System

**DOI:** 10.1371/journal.pone.0097732

**Published:** 2014-05-15

**Authors:** Tao Ren, Jingguo Wang, Qing Chen, Fusuo Zhang, Shuchang Lu

**Affiliations:** 1 College of Resources and Environmental Science, China Agricultural University, Beijing, China; 2 College of Resources and Environment, Huazhong Agricultural University, Wuhan, China; 3 Department of Agronomy, Tianjin Agricultural University, Tianjin, China; University of Maryland, United States of America

## Abstract

With the goal of improving N fertilizer management to maximize soil organic carbon (SOC) storage and minimize N losses in high-intensity cropping system, a 6-years greenhouse vegetable experiment was conducted from 2004 to 2010 in Shouguang, northern China. Treatment tested the effects of organic manure and N fertilizer on SOC, total N (TN) pool and annual apparent N losses. The results demonstrated that SOC and TN concentrations in the 0-10cm soil layer decreased significantly without organic manure and mineral N applications, primarily because of the decomposition of stable C. Increasing C inputs through wheat straw and chicken manure incorporation couldn't increase SOC pools over the 4 year duration of the experiment. In contrast to the organic manure treatment, the SOC and TN pools were not increased with the combination of organic manure and N fertilizer. However, the soil labile carbon fractions increased significantly when both chicken manure and N fertilizer were applied together. Additionally, lower optimized N fertilizer inputs did not decrease SOC and TN accumulation compared with conventional N applications. Despite the annual apparent N losses for the optimized N treatment were significantly lower than that for the conventional N treatment, the unchanged SOC over the past 6 years might limit N storage in the soil and more surplus N were lost to the environment. Consequently, optimized N fertilizer inputs according to root-zone N management did not influence the accumulation of SOC and TN in soil; but beneficial in reducing apparent N losses. N fertilizer management in a greenhouse cropping system should not only identify how to reduce N fertilizer input but should also be more attentive to improving soil fertility with better management of organic manure.

## Introduction

Soil organic matter plays a key role in soil biological and chemical processes, and changes in soil organic matter strongly influence soil N turnover because of the importance of available C for microbial immobilization [1]-[3]. Soils with higher organic matter contents may immobilize more N and reduce N loss to the environment. Otherwise, the depletion of available C will cause more rapid N turnover and losses [4]. In addition, changes in N availability can also alter soil C turnover [5]. There is no doubt that higher crop production in response to mineral N fertilizer application results in greater root exudates and more crop residues, thereby enhancing SOC sequestration in agricultural soils [6]. In addition increasing N fertilizer application can stabilize organic matter [7] and retard the mineralization of older soil organic matter [8]. N fertilization plays a positive role in enhancing the SOC [7], [9]-[12]. However, the addition of N fertilizer has also been reported to have a negative or no effect on SOC accumulation [13]-[17]. Changes in the decomposability of fresh plant litter and soil organic matter fractions, the stability of soil aggregates, and/or shifts in the microbial community can be used to explain the decreases in SOC attributed to N fertilizer addition [13], [15]. Therefore, achieving a better understanding of the interaction between N fertilizer and SOC in agricultural soils is essential for maximizing SOC storage and minimizing potential N losses.

Intensive vegetable production systems in northern China differ from other ecosystems in which excessive nutrients and water are applied, which far exceed the resources needed for vegetable growth [18]-[19]. As shown in previous studies [20]-[21], these practices have resulted in serious N losses to the environment. Therefore, more work was done to understand how to reduce N fertilizer input with optimal N and irrigation strategies, along with catch crops in the intensive greenhouse vegetable cropping system [22]-[24]. Nevertheless, a recent survey of the largest greenhouse vegetable production region in northern China showed that the soil C/N ratio in greenhouse soils was lower than that of the adjacent open field soils because of the high accumulation rate of soil N as a result of an excessive N input [25]. The low soil C/N ratio implied that the C levels were insufficient for the cropping system, which would limit N immobilization by soil microorganisms [2], [26]-[27] and may lead to high N losses [28]. This finding shows that not only optimal N fertilizer management is needed to be studied, but also the soil organic matter content must be improved to enhance soil N retention capacity, which will reduce N losses to the environment and improve N use efficiency.

In a conventional greenhouse vegetable planting system, most of the plant residues are removed at harvest out of fear of infecting the next crop with fungi and other pathogens. Large amounts of organic manure with a low C/N ratio, such as poultry manure and pig manure, are the main soil carbon supplements in the greenhouse vegetable cropping system in northern China [29]. Excessive N fertilizer application is also a significant characteristic of this cropping system. However, it is unclear how the continuous application of poultry manure and excessive mineral N influences the soil organic matter and total N. Whether lower optimized N fertilizer inputs will alter the accumulation of soil organic matter and total N in the greenhouse field? A greenhouse tomato experiment into which different N management strategies were introduced was conducted from 2004 to 2010 in Shouguang county, the largest greenhouse vegetable production region in northern China. In contrast to common farming practice, optimal N management could reduce mineral N fertilizer input by 72% without decreasing the fruit yield [24]. In this experiment, different types of organic manure, including conventional dry chicken manure and wheat straw, were applied. This environment provided an opportunity to gain a better understanding of the influence of organic manure together with mineral N inputs on the SOC and total N pools, and determine if reducing mineral N fertilizer input will alter SOC and TN accumulation. Moreover, it provided an opportunity to analyse the influence of changes in the SOC pool on N losses in the greenhouse vegetable cropping system. Evaluating this system will be of great assistance in improving management practices for maintaining soil fertility and productivity while minimizing potential N losses from the high-input greenhouse vegetable cropping system.

## Materials and Methods

### Ethics statement

No specific permits were required for these field studies. No specific permissions were required for these locations/activities because they were not carried out on privately owned or protected areas. The field studies did not involve endangered or protected species.

### Site description and crop management

**Figure 1:**
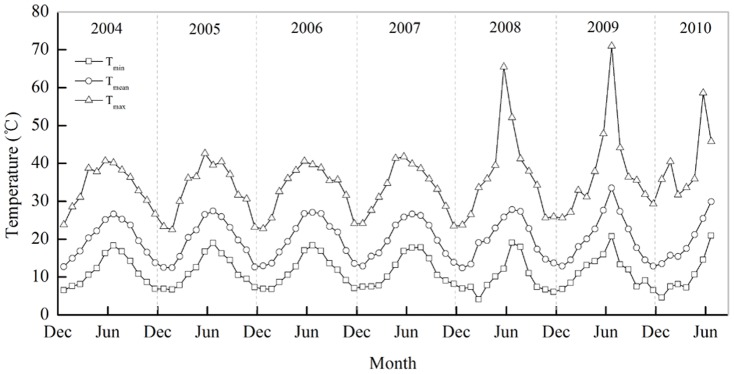
The monthly mean temperature, minimum and maximum temperature inside greenhouse from 2004WS to 2009AW in the year-round greenhouse tomato planting system in Shouguang, northern China.

The experiment was established in February 2004 in a traditional unheated commercial solar greenhouse (84×8.5 m) in Luojia (36°55′N, 118°45′E), Shouguang, northern China. The greenhouse was constructed from a vertical clay wall and covered with polyethylene film throughout the year. Thus the air temperature inside greenhouse is higher than in outside. The monthly mean temperature, minimum and maximum temperature inside greenhouse from 2004 to 2010 was showed in Figure 1. Groundwater was used for irrigation with an average of 573 mm over 11 applications for every growing season. During construction in 1999, surface soil in the greenhouse was used to build the clay wall. The silt loam that remained in the field was approximately 60 cm lower than the open field. Chicken manure was applied at 30 t DW ha^-1^season^-1^ for the first 2 years to improve soil fertility. After 2001, chicken manure application was reduced to 15 t DW ha^-1^ season^-1^ until the experiment was established in 2004. According to FAO classification, the soil at the outset of the trial had 637 g sand kg^-1^, 323 g silt kg^-1^ and 40 g clay kg^-1^ from 0 to 0.1m soil depth, 620 g sand kg^-1^, 335 g silt kg^-1^ and 45 g clay kg^-1^ from 0.1 to 0.3 soil depth, 662 g sand kg^-1^, 301 g silt kg^-1^ and 37 g clay kg^-1^ from 0.3 to 0.6 soil depth, respectively.

Tomato (*Lycopersicon esculentum* Mill.) has been the sole crop since the construction of the greenhouse in 1999. There were two cropping seasons per year, namely a winter-spring (WS) and an autumn-winter (AW) tomato crop. For the WS season, 4-week-old tomato seedlings were transplanted by hand into double rows in the middle of February, harvesting was completed in the middle of June. After a 2-month fallow period, the second (AW) crop was transplanted in early August and the final harvest was taken the following January. The tomato vines were removed from the greenhouse after each final harvest to reduce the infecting of disease carry over into the next crop.

### Treatments

From February 2004 the treatments included (1) **CK**, control treatment, where neither organic manure nor mineral fertilizer N was applied. The N from irrigation water was the important source of N input in the CK treatment, ranging from 25 to 177 kg N ha^-1^ season^-1^ with an average of 102 kg N ha^-1^ season^-1^. The large variation in N input from irrigation water across different growing season was due to different amount of irrigation water and different concentration of NO_3_^—^N in irrigation water. (2) **MN**, organic manure treatment, only organic manure was broadcast as a basal fertilizer with no mineral N fertilizer applied. Organic manure was bought from different poultry farms every growing season and C and N content of chicken manure were different across different growing season. From 2004WS to 2006WS only dry chicken manure was used; and the application rates of dry chicken manure were 8, 11, 8, 11 and 5 t ha^-1^ season^-1^, with an average of 271 kg N ha^-1^ season^-1^. During the autumn-winter season in 2006 (2006AW) and onward, additional chopped wheat straw (1-5 cm long) was added to the soil together with dry chicken manure. From 2006AW to 2009AW the application rates of dry chicken manure were 8, 8, 8, 8, 8, 10 and 8 t ha^-1^ season^-1^; and the rates of wheat straw were 2, 2.5, 4, 2, 4, 4 and 4 t ha^-1^ season^-1^, with an average of 22 kg N ha^-1^ season^-1^. The treatment was also labeled as “MN+S” instead of “MN”. (3) **CN**, conventional N treatment, organic manure was applied as in the MN treatment (except in 2006AW). N fertilizer was applied as a side-dressing at a rate of 120 kg N ha^-1^ on 4-6 occasions based on local farmers normal management practice which depended on the weather conditions, tomato cultivar and growth stage. The average mineral N fertilizer input was 635 kg N ha^-1^ season^-1^. From 2006AW, the CN treatment plot was split into CN and CN+S sub-treatments with plot sizes of 21.8 m^2^ and 32.8 m^2^, respectively. For the CN sub plot only dry chicken manure was applied; and for the CN+S treatment both wheat straw and dry chicken manure were incorporated. The chicken manure and wheat straw were applied at the same rates and timings as in the MN treatment. The mineral N fertilizer application was the same as for the original CN plot. (4) **RN**, reduced N treatment, chicken manure was applied at the same rate as in the MN and CN treatment, and mineral N fertilizer was applied as a side-dressing based on an N target value and soil mineral N content in the root zone (0-30 cm soil layer) for different growth stages from 2004WS to 2007WS. The equation used was as follows:
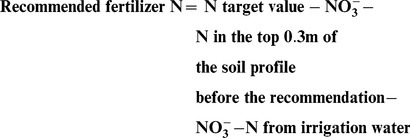
(1)N target value is calculated from crop N uptake, the necessary soil N_min_ residue and soil net mineralization [30], which reflects the synchronization of crop N requirement and soil N supply. Here the initial N target values were 300 kg N ha^−1^ for the side-dressing at each stage of fruit cluster development in 2004. From 2005 the target values from transplanting to the third cluster growth stage were changed to 250 and 200 kg N ha^−1^ for the fourth cluster to the end of harvest in the WS seasons. In the AW season the N target value from transplanting to the fourth cluster growth stage was changed to 200 kg N ha^−1^, with 250 kg N ha^−1^ for the fifth and sixth cluster growth stages [24]. Considering crop N uptake in different growth period and soil N supply, N from irrigation water, only 2-4 side-dressing events were applied according to the differences between N target values and soil N_min_ content in the root zone. From 2007AW the N recommendation was simplified based on the experiences of preceding years. Three or four side-dressing events with an interval of 7–10 days at a rate of 50 kg N ha^−1^ were required in April and October. The more detail introduction of optimized N management was reported by Ren et al [24]. Compared with the CN treatment, 71.3% of mineral N fertilizer was reduced without influencing fruit yield, with an average of 182 kg N ha^-1^ season^-1^. From 2006AW, the RN treatment plot was split into RN and RN+S sub-treatments with the same straw amendments and plot sizes as in the CN treatment. During 2006AW and 2007WS growing season, the mineral N fertilizer application rates of RN and RN+S treatment were determined based on the N target values and soil N_min_ content in the root zone before side-dressing. There were litter differences on mineral N fertilizer application rates between RN and RN+S treatment. Since 2007AW, the mineral N fertilizer application was the same for RN and RN+S treatment.

All treatments were set up in a randomized block design with three replicates. Plots were separated for each other by plastic film at a depth of 30 cm. The exogenous C and N inputs are shown in Table 1. The average C input from chicken manure was 2779 kg C ha^-1^ season^-1^, with a range of 1114 to 5445 kg C ha^-1^ season^-1^. Beginning in 2006AW, an average of 1119 kg wheat straw C ha^-1^ season^-1^ was supplied, and the total C input was as high as 3483 kg C ha^-1^ season^-1^. The N sources included mineral N fertilizer, organic manure and irrigation water. In the CK treatment, the N from irrigation water was the only source of N input, with an average of 102 kg N ha^-1^ season^-1^. The average exogenous N inputs for the MN, RN and CN treatments were 354, 527 and 984 kg N ha^-1^ season^-1^, respectively.

**Table 1:** Exogenous C and N inputs in the greenhouse tomato production system in Shouguang, northern China (kg ha^-1^ season^-1^).

Growing season	C^1^						N^1^					
	CK	MN(MN+S)^3^	RN	RN+S	CN	CN+S	CK	MN(MN+S)	RN	RN+S	CN	CN+S
2004WS^2^	0	2860	2860	-	2860	-	56	316	644	-	1186	-
2004AW^2^	0	5445	5445	-	5445	-	118	478	638	-	1198	-
2005WS	0	3476	3476	-	3476	-	54	370	497	-	1000	-
2005AW	0	3902	3902	-	3902	-	177	435	636	-	1155	-
2006WS	0	1114	1114	-	1114	-	165	327	465	-	927	-
2006AW	0	4095	3423	4095	5135	5807	133	456	651	671	1200	1212
2007WS	0	2258	1698	2258	1698	2258	144	308	509	486	838	848
2007AW	0	4806	3406	4806	3406	4806	82	417	492	517	872	897
2008WS	0	2110	1606	2110	1606	2110	25	180	321	330	771	780
2008AW	0	3052	1606	3052	1606	3052	39	202	382	402	782	802
2009WS	0	4205	2592	4205	2592	4205	135	441	605	641	1105	1141
2009AW	0	3857	2214	3857	2214	3857	99	322	489	523	769	802
Average	0	3432	2779	3483	2921	3728	102	354	527	510	984	926

1 C input from chicken manure and wheat straw; N input from mineral fertilizer, organic manure and irrigation water;

2 WS: winter-spring growing season, AW: autumn-winter growing season;

3 Since 2006AW chopped wheat straw and dry chicken manure was broadcast as a basal fertilizer and the treatment was labeled as “MN+S” instead of “MN”;

Urea was the main mineral N fertilizer. All plots received P_2_O_5_ as calcium monophosphate (12% P_2_O_5_) and K_2_O as potassium sulfate (50% K_2_O) during each growing season. The average input during the past 12 growing seasons were 350 kg P_2_O_5_ ha^-1^ and 563 kg K_2_O ha^-1^ per season.

### Soil sampling and analysis

#### Soil organic carbon and total N concentrations

Three soil cores (3.5 cm in diameter) were taken from each plot to a depth of 0.6 m and subdivided into 0-0.1, 0.1-0.3 and 0.3-0.6 m increments on April 18, 2004, and January 20, 2010. Fresh soil cores were taken to the lab immediately, mixed thoroughly to provide a composite sample from each plot, sieved through a 2 mm mesh and then air-dried and stored in plastic bottles. After removing any carbonates with 1 M HCl, the SOC and total N concentrations were determined using a C and N analyzer (vario MACRO CN, Elementar, Germany).

At the same time, soil bulk density in different soil layers was measured by the cutting ring method. In 2004, three samples were collected from the whole greenhouse and the average soil bulk densities were 1420 kg m^-3^, 1450 kg m^-3^ and 1480 kg m^-3^ for the 0-10 cm, 10-30 cm and 30-60 cm soil layers, respectively. The bulk density of each plot monitored in 2010 is shown in Table 2 and no significant differences were observed among the different treatments.

**Table 2:** Soil bulk density in the soil profile in Jan. 2010 in a year-round greenhouse tomato planting system in Shouguang, northern China (kg m^-3^).

Treatment	Soil layer (cm)		
	0-10	10-30	30-60
CK	1333±41	1545±70	1589±63
MN(MN+S)^1^	1296±118	1548±79	1586±56
RN	1301±82	1502±30	1460±42
RN+S	1285±35	1508±126	1546±87
CN	1227±213	1584±129	1512±127
CN+S	1283±43	1493±114	1542±132
Average	1288	1530	1539

1 Since 2006AW chopped wheat straw and dry chicken manure was broadcast as a basal fertilizer and the treatment was labeled as “MN+S” instead of “MN”;

#### Soil organic carbon fraction

Changes in the quantity and quality of the SOM pool are generally difficult to detect in the short term following agricultural management. Labile and recalcitrant SOM separated by different methods provide a more sensitive indicator for evaluating the effect of different management strategies on SOM dynamics [31]-[33]. Here, soil organic carbon fractionation procedures were carried out as described by Blair et al [31]. Air-dried soil samples containing approximately 15 mg of C were oxidized with 25 mL of 333 mmol L^-1^ KMnO_4_ for 1 h at 25°C on a shaker at 180 rpm. The samples were then centrifuged, diluted and spectrophotometrically measured at 565 nm. The oxidized carbon was considered labile C, and the remainder represented the non-labile C.

#### Apparent N losses

The nutrient balance is often used to estimate potential environmental risks [34]-[35]. The apparent N losses were calculated according to Equation (2), as described by Ren et al [24], as follows: 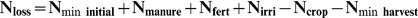
(2)where N_loss_  =  apparent N loss, N_min initial_  =  soil N_min_ content at 0-0.6 m before transplanting, N_manure_  =  total N input from organic manure, N_fert_  =  N from mineral fertilizer, N_irri_  =  NO_3_^—^N from irrigation water, N_crop_  =  total N uptake by tomato aboveground parts, and N_min harvest_  =  soil N_min_ content at 0-0.6 m at the end of the harvest.

The parameters were calculated as follows:

Three soil cores (3.5 cm in diameter) were collected from each plot at a depth of 0.6 m and then subdivided into 0-0.3 m and 0.3-0.6 m increments before transplanting and at the end of the harvest for each season. Fresh soil cores were mixed thoroughly to give a composite sample from each plot and then passed through a 2 mm sieve. Next, 12 g subsamples were weighed and extracted by shaking with 100 mL of 1 mol L^-1^ KCl for 1 h. The extract was stored at -18°C until an analysis of the NO_3_^—^N and NH_4_^+^-N concentrations could be carried out with a continuous flow analyzer (TRAACS Model 2000). The water content of the soil samples was also gravimetrically determined to calculate the soil N_min_ (NO_3_^—^N+NH_4_^+^-N) content on a dry matter basis. The soil bulk density was used to convert the mineral N in mg per kg of soil to kg per hectare.

The input of N from irrigation water was determined by recording the amounts applied, and water samples were collected during each irrigation event over the entire growing season. The samples were stored frozen until NO_3_^—^N and NH_4_^+^-N analysis.

Plant samples were collected from each plot at the end of the harvest; divided into leaves, fruit, stems and roots; and weighed before and after drying at 70°C for 48 h. The dried shoots were ground before determining the total N, which was conducted using a modified Kjeldahl method with salicylic acid. N uptake was calculated as the product of dry matter and total N concentration in different parts.

#### Data analysis

Analysis of variance (*ANOVA*) was used to determine the significance of treatment effects based on a randomized complete block design. Multiple comparisons of mean values were performed using either Duncan's multiple range tests or Fisher's protected least significant difference (*LSD*) test at the 0.05 level of probability. Statistical analysis was performed using version 6.12 of the SAS software package (SAS Institute Inc., Cary, NC).

## Results

### Soil organic carbon and total N concentrations

**Figure 2:**
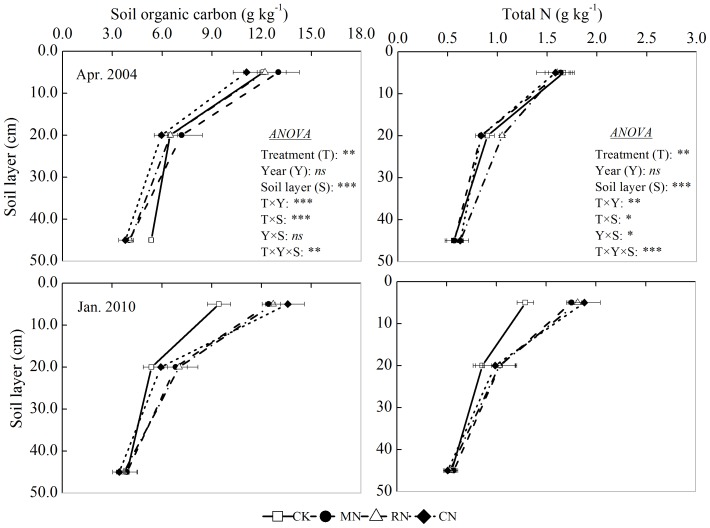
The distribution of soil organic carbon and total N concentrations in the soil profile with different N treatments in the year-round greenhouse tomato planting system in Shouguang, northern China. Note: *, ** and *** indicate significant differences at *P*<0.05, *P*<0.01 and *P*<0.001, ns denotes no significant difference.

Soil organic carbon (SOC) and total soil N (TN) concentrations in soil profiles to a depth of 60 cm are shown in Figure 2. The highest SOC concentration was seen in the 0-10 cm layer, below which it decreased for all treatments. In April of 2004, the SOC of the soil profile was similar across all N treatments, except there were some variations in the 30-60 cm layer associating with uneven soil fertility. After 6 years, SOC in the CK treatment was significantly lower than that of the other treatments in the 0-10 cm soil layer. However, there were no significant differences among the other treatments. In the 10-30 cm layer, SOC differed significantly between the RN and CN treatments. In comparison to the initial value, SOC decreased significantly in all CK treatment layers after 6 years of cultivation, but the only significant change in SOC for the CN treatment was an increase in the 0-10 cm layer.

Patterns in total N in the soil profile followed a similar pattern to that of the SOC (Figure 1). In January 2010, soil TN in the CK treatment was significantly lower than that of the other treatments in the 0-10 cm layer. In addition, no significant differences were observed among the MN, RN and CN treatments. The TN values did not show significant differences across N treatments in the 10-60 cm layer in 2010.

### Soil organic carbon and total N pool

Total SOC and TN pool in the profile above 60 cm were 46.6-55.1 t C ha^-1^ and 6.8-8.3 t N ha^-1^, respectively (Table 3). After 6 years of cultivation, approximately 13.1 t C ha^-1^ and 2.2 t C ha^-1^ were lost from the CK and MN treatments, respectively. The decreased bulk density was mainly attributed to the decreased SOC in the MN treatment (Table 2). The SOC pool increased to 0.8 t C ha^-1^ and 1.7 t C ha^-1^ in response to the RN and CN treatments, respectively. For the N pool, the only reduction occurred in the CK treatment. Approximately 0.63 t N ha^-1^ was lost over the last 6 years. Average accumulation rates of SOC and TN in the 0-60 cm layer were -2.17, -0.37, 0.14 and 0.28 t C ha^-1^ a^-1^ and -0.10, 0.14, 0.00 and 0.06 t N ha^-1^ a^-1^ for the CK, MN, RN and CN treatments, respectively. The addition of straw over 4 years made little difference to SOC and TN concentration

**Table 3:** Distribution of the soil organic C and N pools in the soil profile of a greenhouse tomato production system in Shouguang, northern China.

		SOC pool (t ha^-1^)						N pool (t ha^-1^)					
		CK	MN (MN+S)^2^	RN	RN+S	CN	CN+S	CK	MN (MN+S)^2^	RN	RN+S	CN	CN+S
Apr-04	0-10 cm	17.2±0.2	18.5±1.8	17.3±1.8	-	15.7±1.2	-	2.36±0.11	2.32±0.17	2.27±0.17	-	2.25±0.27	-
	10-30 cm	18.8±1.3	20.8±3.7	18.8±2.0	-	17.3±1.3	-	2.61±0.21	2.41±0.15	3.04±0.07	-	2.43±0.05	-
	30-60 cm	23.7±0.6	17.8±0.5	18.1±0.9	-	16.8±1.8	-	2.50±0.39	2.52±0.33	2.79±0.37	-	2.78±0.14	-
Jan-10	0-10 cm	12.2±0.9	16.0±0.5	16.4±0.6	17.9±0.9	17.5±1.3	17.6±1.0	1.66±0.11	2.26±0.06	2.34±0.05	2.42±0.09	2.4±0.21	2.54±0.12
	10-30 cm	16.5±1.5	20.9±2.2	21.6±3.5	17.2±1.2	18.2±1.3	18.6±2.1	2.63±0.28	3.17±0.52	3.15±0.50	2.70±0.23	3.02±0.61	2.95±0.18
	30-60 cm	18.0±2.8	18.0±2.9	17.1±0.1	17.2±1.5	15.8±1.8	18.7±0.7	2.55±0.22	2.64±0.20	2.62±0.14	2.68±0.06	2.35±0.19	2.86±0.41
△(0-60 cm)^1^		-13.1±4.5	-2.2±1.0	0.8±6.1	-	1.7±3.6	-	-0.63±0.50	0.82±0.27	0.01±0.74	-	0.34±0.47	-
Accumulation rate (t ha^-1^ a^-1^)		-2.17	-0.37	0.14	-	0.28	-	-0.10	0.14	0.00	-	0.06	-

1 △(0-60 cm)  =  SOC (N) pool_2004_- SOC (N) pool _2010_;

2 Since 2006AW chopped wheat straw and dry chicken manure was broadcast as a basal fertilizer and the treatment was labeled as “MN+S” instead of “MN”;

### Soil organic carbon fractions

**Figure 3:**
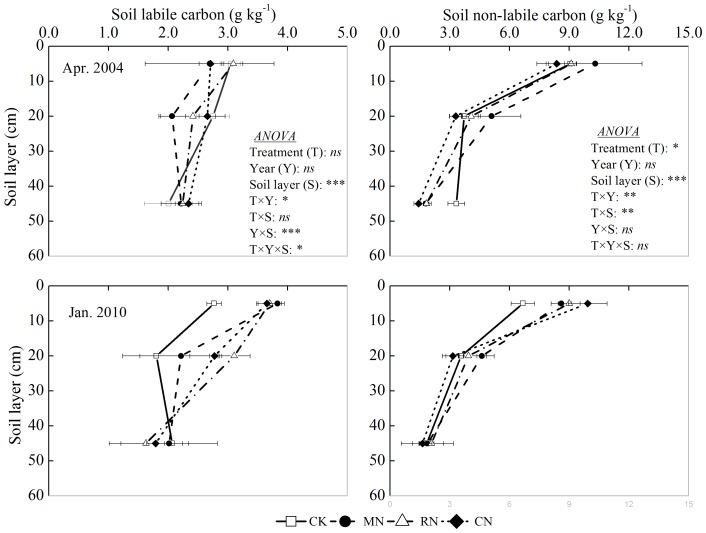
The distribution of the soil labile and non-labile carbon concentrations in the soil profile with different N treatments in the year-round greenhouse tomato planting system in Shouguang, northern China. Note: Note: *, ** and *** indicate significant differences at *P*<0.05, *P*<0.01 and *P*<0.001, ns denotes no significant difference.

Figure 3 shows the distribution of labile and non-labile carbon in different soil layers. In April 2004, the labile C concentrations were similar across all N treatments in the same layer. However, in January 2010, the soil labile C concentration in the CK treatment was significantly lower than that of the other treatments in the 0-30 cm layer. Compared with the initial values in 2004, the soil labile C concentration in the CK treatment did not decrease significantly according to paired-sample T test; however, labile C increased significantly in the RN and CN treatments for the 0-10 cm layer.

Changes in the concentration of the non-labile C fraction in the soil profile were similar to those of the SOC. In January 2010, the soil non-labile C concentration in the 0-10 cm layer increased as the N application increased. When compared with April 2004, it decreased significantly in the CK treatment. Nevertheless, it increased significantly in the CN treatment. For all other treatments, no significant changes were observed in the soil profile.

### Apparent N losses

**Figure 4:**
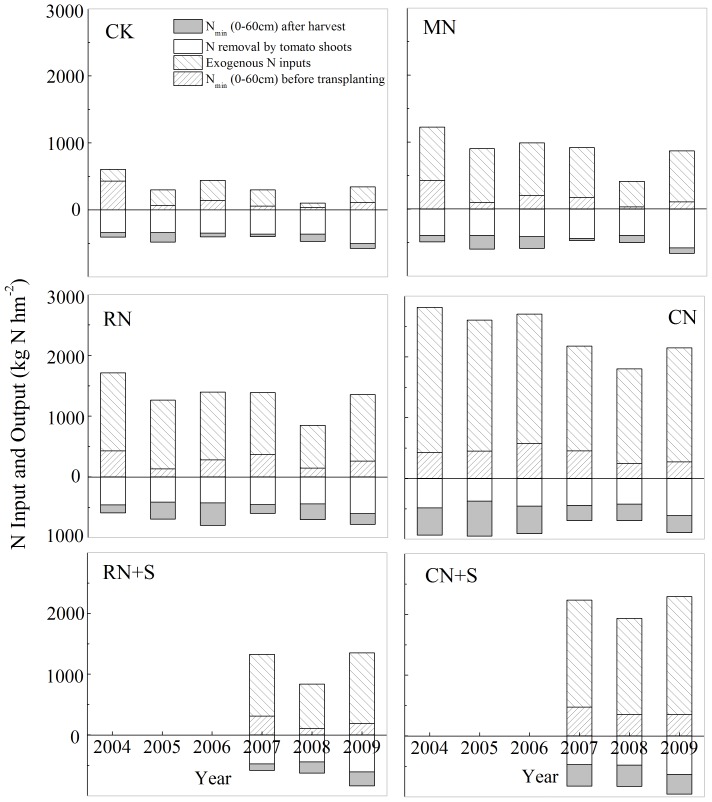
Apparent N loss with different treatments in the year-round greenhouse tomato planting system from 2004 to 2009 in Shouguang, northern China.

Figure 4 shows the annual apparent N losses from 2004 to 2009 as obtained estimated from the nitrogen balance. For the CN treatment, the average annual mean apparent N losses were as high as 1529 kg N ha^-1^ a^-1^, accounting for 77% of the annual exogenous N input. A significant decrease in the annual apparent N losses of 633 kg N ha^-1^ a^-1^ occurred in the RN treatment because the rate of N fertilization had been reduced to less than one-third of that in the CN treatment. For the CK treatment, soil N and nitrate in the irrigation water were the major sources of N, which were less input than N removed by plant uptake, resulting in a negative N balance. These changes could indirectly explain the decreased TN in the CK treatment. Although the straw treatments led to the addition of C, the apparent N losses from treatments with and without straw amendments did not differ significantly. The N surpluses were 490 and 1285 kg N ha^-1^ a^-1^ N for RN+S and CN+S, respectively.

### Interactions between C and N in soil

**Figure 5:**
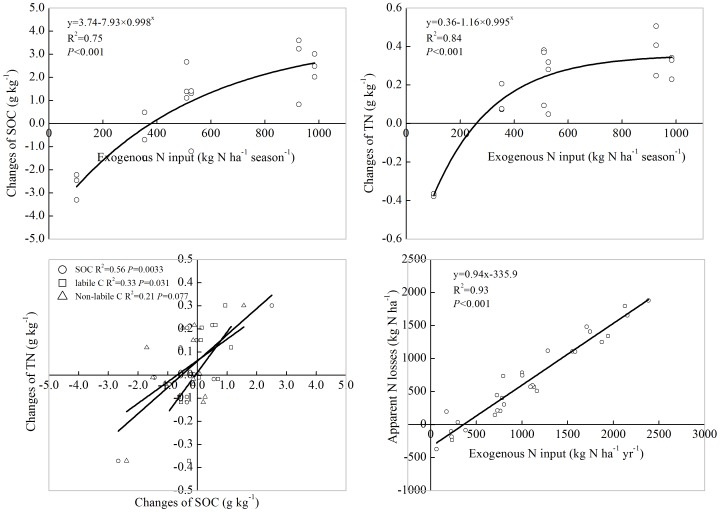
The relationship between the changes in SOC and TN concentration after 6 years of cultivation, as well as annual apparent N losses and the average N inputs in the year-round greenhouse tomato planting system in Shouguang, northern China.

Figure 5 shows the relationship between the changes in SOC and TN concentration after 6 years of cultivation, as well as annual apparent N losses and average N inputs. The SOC and TN concentrations increased in response to N addition but showed a negative response to excessive N application (Figure 5a, b). The total N in the soil did not increase linearly with the increase in applied mineral N, which might be explained by changes in the SOC and its fractions (Figure 5c). The minor changes in SOC in response to the current type and amount of organic manure application, limited N storage in soil and N was close to saturation. With the increase in fertilizer N application, the apparent N losses linearly increased and conventional N fertilizer management caused the highest apparent N losses (Figure 5d).

## Discussion

### Effect of N fertilizer on SOC

Greater root exudates and more crop residues in response to mineral N fertilizer application were the dominant reasons why N fertilizer application improved the SOC [6] ^.^ In this experiment, although 71.3% of mineral N fertilizer was cut down in the RN treatment compared with the CN treatment, there were no significant differences on fruit yields and plant biomass between the CN and RN treatment [24]; root exudates were presumed to be similar between these two treatments. Besides, in our greenhouse system crop residues were removed from the greenhouse at harvest because of the risks of disease carryover, so organic manure was the major C supplement. The C input from organic manure was the same for the CN and RN treatments in the same growing season (Table 1). Moreover, no significant changes in the root C/N ratio (data not shown), soil microbial community [36] and soil organic matter fractions (Figure 3) between the RN and CN treatments were found. Thus, no significant differences in SOC pool between the CN and the RN treatment were observed in this experiment, which was similar to other work [14], [17]. Apparently, N was not limiting factor in this cropping system and reduced mineral N input would not alter SOC and TN accumulation. All these findings indicate that when organic manure is used, optimizing N fertilizer input over a continuous 6-year period would not affect the SOC or TN contents in the greenhouse vegetable cropping system; but it was helpful in reducing the environmental risks without influencing fruit yields (Figure 4).

### Effect of organic manure on SOC

Organic manure application brought lots of N, with the average of 252 kg N ha^-1^. In contrast to the CK treatment, significant increments on fruit yields were achieved; yet there were no differences on fruit yields and plant biomass between MN+S treatment and RN, CN treatment in most growing seasons [24], demonstrating that N from organic manure was important N source for crop growth and excessive mineral N fertilizer application was wasteful without considering N from organic manure. In addition, Organic manure application is considered to be a consistent method for maintaining soil fertility over the long-term [10], [37]. In this unique production system, the major source of organic carbon is organic manure. If the input of organic manure is excluded, only 44-146 kg C ha^-1^ season^-1^ from residual roots is incorporated [38]. If no organic manure is applied, SOC concentration, especially for stable organic carbon, will decline significantly (Figure 2, 3). These results are similar to those of another long-term greenhouse tomato experiment [39].

Before the start of this experiment, about 210 t ha^-1^ chicken manure had been applied across the whole greenhouse during 1999-2003; and the high organic manure application might build higher soil organic carbon pool in a short time. Therefore, in contrast to the treatments with organic manure application, there was the lack of a big difference on SOC in the CK treatment for the next 6 years. As well, no observable changes in soil organic C was found over 6 years of successive chicken manure applications. Whether it implied the soil organic carbon pool in our study was saturated? Although the coarse-textured soil has lower capacity for C and N stabilization, the saturated soil organic carbon pool in 0-30cm soil layer could be high to 75.6-96.8 t ha^-1^ according to Hassink [40] and Six's C-saturation model [41]. These values were higher than it reported in our study, indicating that soil organic carbon could be improved with optimum management. Quantity and quality of input organic manure significantly influenced soil organic C dynamics. In contrast to farmers' normal manure application, the application rate of chicken manure in the experiment was lower, ranging from 8 t ha^-1^ season^-1^ to 12 t ha^-1^ season^-1^. Whether SOC content would be enhanced with the organic manure application rate increase? Indeed, high rates of organic manure application were conducive to enhanced SOC and SOC fractions [42]-[43]. Ge et al [39] demonstrated that it would take 10-15y with 75 t ha^-1^ a^-1^ of horse manure to increase the soil organic matter content from 24 g kg^-1^ to 30-40 g kg^-1^ in the greenhouse vegetable soil. However, environmental pressures associating with excessive application of organic manure were serious [44]; and in Europe the applications of organic manure are restricted to not exceed 170 kg N ha^-1^ y^-1^ by the legislation. In our experiment N input from organic manure averaged 252 kg N ha^-1^ season^-1^, with 335 kg N ha^-1^ y^-1^ apparent N loss. Obviously it will not be an effective way to enhance SOC relying on the increments of organic manure application rate in greenhouse vegetable cropping system. Changing the type of organic manure input might be an important way to heighten SOC. For chicken and pig manure with high proportions of water-soluble C and easily biodegradable organic compounds, approximately 45-62% of C is evolved as CO_2_-C within 30 days [45]-[46]. Plaza et al [47] reported a significant decrease in the total organic C in soils amended with pig manure slurry. However, manure with a greater ratio of C/N, or high content of recalcitrant C could reduce mineralization of bio-labile compounds, thereby enhancing soil organic matter [48]-[49]. Long term field experiments showed that the benefits in SOC content were higher from application of rice straw and compost than that from pig manure [42], [50].

To improve soil organic carbon content, wheat straw was added from in 2006AW. However, no significant increase in the SOC or the SOC fraction was observed after 4 years of cultivation. This result was similar to that of Antil's study [51], which found that the SOC in bulk soil decreased or was not affected by a slurry + straw treatment in both fallow and cropped plots, even after 28 and 38 yrs. According to the mechanisms of real and apparent priming effects [52], it is assumed that when chicken manure and wheat straw are applied together, microorganisms may first use the C from chicken manure to activate the microbial community; however, when the easily decomposed organic carbon is consumed, activated microorganisms will use the wheat straw carbon. Most of added straw carbon is then utilized by microorganisms, perhaps explaining why there was no effect on SOC when chicken manure and wheat straw were incorporated together. The short duration might be another important reason why no differences were seen. Additional long-term studies should be conducted to determine if the application of a mixture of wheat straw and chicken manure is an effective method of enhancing soil organic carbon in the greenhouse vegetable cropping systems over the long run. Overall, developing an optimum organic manure management system to enhance soil fertility is now one of the important issues in greenhouse vegetable cropping systems in China. In comparison to increasing the application rate, shifting the type of organic manure from pig and chicken manure to manure with a wider ratio of C/N or high content of recalcitrant C may be more practical for enhancing soil organic matter in greenhouse vegetable cropping system.

### Effect of soil organic carbon content on the fate of N

Similar to the results from 2004 to 2007 [24], approximately 77% of the exogenous N input was surplus in the CN treatment. With the exception of immobilized N in soil clays, N leaching [53] and N_2_O emissions [21] were the major N loss processes in these vegetable cropping systems. Furthermore, warm and moist conditions with sufficient available nitrate and labile carbon can lead to denitrification loss, which might also be an important N loss process [54]. In any case, an excessive N surplus would lead to high potentially N losses. Therefore, most studies on the prevention of N losses have focused on reducing the N input and on irrigation strategies [24], [55] and catch crops [56]. In the experiment, the rate of N fertilization in the RN treatment had been reduced to less than one-third of that in the CN treatment and apparent N losses were also decreased. Other than that, SOC plays an important role in regulating soil N turnover and the improvement of SOC is beneficial to increase potential rates of N immobilization and reduce N losses [57]. Yang et al [58] showed that the rate of absolute N change increased linearly with changes in the size of the C pool change and organic N capital was determined by long-term carbon sequestration. A similar tendency was observed in the present study (Figure 3c). In our experiment, SOC concentration was not increased even though high amounts of organic manure were applied during a 6 year period; Changes in organic N were similarly limited. Moreover, the mineralization rate of total organic N might be greater than the retention of exogenous N to soil organic N pool because of the great amount of organic manure applied within several years before the experiment started. Thus more exogenous N was lost to the environment. The low soil C/N ratio indirectly revealed that there was insufficient C in the cropping system would limiting N immobilization by soil microorganisms [2], [26] and leading to high N losses [28]. Therefore, improving soil organic matter content and enhancing potential rates of soil N immobilization according to optimal organic manure management was crucial, as well reducing N fertilizer input, to lower N losses in greenhouse vegetable cropping system.

## Conclusion

Organic manure represents a major organic C source in conventional greenhouse vegetable cropping systems in China. Without additions of organic manure, SOC, particularly stable C is likely to decline. However, no significant increment in SOC was observed with the addition of high amounts of low C/N ratio organic manure or plant residues. Shifting the type of manure from chicken manure to manures with a wider ratio of C/N, or high content of recalcitrant may be more effective in enhancing soil fertility in greenhouse vegetable production. On the basis of organic manure application, optimized N fertilizer inputs according to root-zone N management did not influence the accumulation of SOC and TN in soil; but beneficial in reducing apparent N losses.

The SOC concentration was a dominant limiting factor for soil total N enhancement. Given the current type and quantity of organic manure application, the SOC concentration was unchanged and most applied N was in excess and was lost to the environment. Therefore, integrating nutrient management, including optimized N fertilizer input, as well as enhanced the soil organic matter content, should be considered to maintain soil fertility and productivity, minimize potential N losses and achieve sustainable development in greenhouse vegetable cropping systems.
